# A New Decomposition of the Graph Laplacian and the Binomial Structure of Mass-Action Systems

**DOI:** 10.1007/s00332-023-09942-w

**Published:** 2023-08-02

**Authors:** Stefan Müller

**Affiliations:** Faculty of Mathematics, University of Vienna, Oskar-Morgenstern-Platz 1, 1090 Vienna, Austria

**Keywords:** Labeled directed graph, Chemical reaction network, Weak reversibility, Complex-balanced equilibrium, Asymptotic stability, Differential inclusion, 05C50, 34D20, 92C45

## Abstract

We provide a new decomposition of the Laplacian matrix (for labeled directed graphs with strongly connected components), involving an invertible *core matrix*, the vector of tree constants, and the incidence matrix of an auxiliary graph, representing an order on the vertices. Depending on the particular order, the core matrix has additional properties. Our results are graph-theoretic/algebraic in nature. As a first application, we further clarify the binomial structure of (weakly reversible) mass-action systems, arising from chemical reaction networks. Second, we extend a classical result by Horn and Jackson on the asymptotic stability of special steady states (complex-balanced equilibria). Here, the new decomposition of the graph Laplacian allows us to consider regions in the positive orthant with given *monomial evaluation orders* (and corresponding polyhedral cones in logarithmic coordinates). As it turns out, all dynamical systems are asymptotically stable that can be embedded in certain *binomial differential inclusions*. In particular, this holds for complex-balanced mass-action systems, and hence, we also obtain a polyhedral-geometry proof of the classical result.

## Introduction

The Laplacian matrix (or graph Laplacian) is a matrix representation of a graph. It can be seen as a discrete version of the Laplace operator defined on graphs. On the one hand, the Laplacian matrix of an *undirected* graph, its spectrum, and its eigendecomposition have a variety of applications ranging from organic chemistry to signal processing and machine learning (Mohar 1991; Merris 1994; Shuman et al. 2013; Belkin and Niyogi 2003). On the other hand, *labeled, directed* graphs underlie dynamical systems ranging from continuous-time Markov processes (linear stochastic models) (Mirzaev and Gunawardena 2013) to mass-action systems (nonlinear deterministic models of chemical reaction networks) (Horn and Jackson 1972).

In the linear setting, the vertices *V* of a simple digraph $G=(V,E)$ represent states, and the edges *E* represent transitions. Moreover, edge labels *k* represent transition rate constants. The dynamical system for a state variable $\psi$ is given by

1 $$\begin{aligned} \frac{\textrm{d} \psi }{\textrm{d} t} = A_k \, \psi , \end{aligned}$$

where $A_k$ is the Laplacian matrix of the labeled digraph $G_k=(V,E,k)$. That is, $(A_k)_{i,j} = k_{j \rightarrow i}$ if there is a transition $(j \rightarrow i) \in E$, $(A_k)_{i,i} = -\sum _{(i \rightarrow j) \in E} k_{i \rightarrow j}$, and $(A_k)_{i,j}=0$ otherwise. (As in chemical reaction network theory, we use the letter *A* for the graph Laplacian and indicate its dependence on the edge labels *k* by a subscript.) The linear system can be called “Laplacian dynamics”, it is equivalent to the stochastic master equation, and it is studied in applications ranging from biochemistry to systems biology (Gunawardena May 2012; Mirzaev and Gunawardena 2013).

In the nonlinear setting, the dynamical system for the species concentrations *x* is given by

2 $$\begin{aligned} \frac{\textrm{d} x}{\textrm{d} t} = Y A_k \, x^Y. \end{aligned}$$

All notations are defined at the end of this introduction, and mass-action systems are introduced in Sect. 3. Here, we motivate Eq. (2) in an informal way. As an example, we consider the chemical reaction ${1 \textsf{X}_1+ 1 \textsf{X}_2 \rightarrow \textsf{X}_3}$ with “stoichiometric” coefficients equal to 1. Under the assumption of mass-action kinetics, its rate is given by $v = k \, (x_1)^1 (x_2)^1$, where $k > 0$ is the rate constant, and $x_1, x_2 \ge 0$ are the concentrations of the species $\textsf{X}_1,\textsf{X}_2$. More abstractly, we can write the reaction as ${y \rightarrow y'}$ with (educt and product) “complexes” $y = (1,1,0,0,\ldots )^\textsf{T}$ and $y'=(0,0,1,0,\ldots )^\textsf{T}$, and we can write its rate as $v = k \, x^y$ with the monomial $x^y := \prod _j (x_j)^{y_j}= (x_1)^1 (x_2)^1 (x_3)^0 (x_4)^0 \cdots$ in the species concentrations $x=(x_1,x_2,x_3,x_4,\ldots )^\textsf{T}$. In a network, an individual reaction ${y \rightarrow y'}$ contributes the summand $k \, x^y \, (y' - y)$ to the dynamical system for *x*, where the reaction vector $y'-y$ captures the consumption of educts *y* and the formation of products $y'$. For the example reaction, $x^y=x_1 x_2$ (as stated above) and $y'-y = (-1,-1,1,0,\ldots )^\textsf{T}$.

Now, we can introduce a mass-action system as a simple digraph $G=(V,E)$, a map *y* (assigning complexes to vertices), and edge labels *k*. In particular, every edge ${(i \rightarrow i') \in E}$ defines a reaction ${y(i) \rightarrow y(i')}$ with rate constant $k_{i \rightarrow i'}$. Hence, the associated dynamical system $\frac{\textrm{d} x}{\textrm{d} t} = \sum _{(i \rightarrow i') \in E} k_{i \rightarrow i'} \, x^{y(i)} \, ( y(i')-y(i) )$ involves a sum over all edges, and every summand is a product of a reaction rate and a reaction vector. Using the Laplacian matrix $A_k$, the right-hand-side can be decomposed as shown in Eq. (2). The matrix *Y* collects the complexes *y*(*i*) for $i \in V$, and the vector of monomials $x^Y$ is defined via $(x^Y)_i = x^{y(i)}$. Altogether, the dynamical system is polynomial. It is determined by the complex matrix *Y* (by stoichiometry) as well as by the Laplacian matrix $A_k$ (by the graph), and chemical reaction network theory studies the interplay of these two matrices to understand dynamics and steady states of mass-action systems, starting from the foundational 1972 papers (Horn and Jackson 1972; Horn 1972; Feinberg 1972) until today.

A steady state $x > 0$ with $A_k \, x^Y=0$ is called a positive *complex-balanced* equilibrium (CBE), also known as vertex-balanced steady state. Indeed, at a CBE, the sum of all “flows” $k_{i \rightarrow i'} \, x^{y(i)}$ *from* vertex *i*/complex *y*(*i*) equals the sum of all $k_{i' \rightarrow i} \, x^{y(i')}$ *to* the latter. As shown by Horn (1972) and Horn and Jackson (1972) in 1972, the existence of a CBE has three important consequences: the components of the graph are strongly connected (the network is “weakly reversible”); all equilibria are complex-balanced and asymptotically stable; and there is a unique equilibrium in every dynamically invariant affine subspace (“stoichiometric compatibility class”). More technically, complex-balanced equilibria are given by binomial equations and have a monomial parametrization.

For symmetric digraphs (“reversible” networks), *detailed-balanced* equilibria are given by binomial equations (by definition). Moreover, the polynomial dynamical system is a sum of binomials. (Just note that every reversible reaction ${y \rightleftarrows y'}$ contributes the summand $(k_{y \rightarrow y'} \, x^y - k_{y' \rightarrow y} \, x^{y'})\, (y' - y)$ to the dynamical system for *x*). We show that this also holds for weakly reversible networks. To this end, we provide a new decomposition of the graph Laplacian, involving an invertible *core matrix*, based on an order on the vertices. Further, we extend the classical result by Horn and Jackson on the asymptotic stability of complex-balanced equilibria. In addition to a Lyapunov function (as in classical proofs), we consider regions in the positive orthant with given *monomial evaluation orders* (and corresponding polyhedral cones in logarithmic coordinates). As it turns out, all dynamical systems are asymptotically stable that can be embedded in certain *binomial differential inclusions*. In particular, this holds for complex-balanced mass-action systems, and hence, we also obtain a polyhedral-geometry proof of the classical result.

### Organization of the Work

In Sect. 2, we provide a new decomposition of the graph Laplacian (for labeled directed graphs with strongly connected components), involving an invertible *core matrix*, based on an order on the vertices. Depending on the particular order, the core matrix has additional properties.

In Sect. 3, we apply the graph-theoretic/algebraic results to mass-action systems. In Sect. 3.1, we demonstrate their binomial structure, and in Sect. 3.2, we introduce *monomial evaluation orders* and corresponding geometric objects (polyhedra and polyhedral cones). In Sect. 3.3, we embed complex-balanced mass-action systems in *binomial differential inclusions* and show that all equilibria of the latter are asymptotically stable, and in Sect. 3.4, we discuss our results.

In “Appendix A”, we provide explicit formulas for the vector of tree constants and the Laplacian matrix, using cycle decomposition. In “Appendix B”, we state auxiliary results used in the new decomposition of the graph Laplacian. In “Appendix C”, we give another proof of the asymptotic stability of complex-balanced equilibria (and the non-existence of other steady states) without using differential inclusions.

**Notation** We denote the positive real numbers by ${\mathbb R}_>$ and the nonnegative real numbers by ${\mathbb R}_\ge$. Throughout the work, we use index notation: for a finite index set *I*, we write ${\mathbb R}^I$ for the real vector space of vectors $x=(x_i)_{i \in I}$ with $x_i \in {\mathbb R}$, and analogously we write ${\mathbb R}^I_\ge$ and ${\mathbb R}^I_>$. (For $I=\{1,\ldots ,n\}$, we have the standard case ${\mathbb R}^I={\mathbb R}^n$.) We write $x>0$ for $x \in {\mathbb R}^I_>$ and $x \ge 0$ for $x \in {\mathbb R}^I_\ge$.

For vectors $x,y \in {\mathbb R}^I$, we denote their scalar product by $x \cdot y \in {\mathbb R}$ and their componentwise (Hadamard) product by $x \circ y \in {\mathbb R}^I$. For $x \in {\mathbb R}^I_>, \, y \in {\mathbb R}^I$, we define the (generalized) monomial $x^y = \prod _{i \in I} (x_i)^{y_i} \in {\mathbb R}_>$, and for $x \in {\mathbb R}^I_>, \, Y \in {\mathbb R}^{I \times J}$, we define the vector of monomials $x^Y \in {\mathbb R}^J_>$ via $(x^Y)_j =x^{y(j)}$, where *y*(*j*) is the column of *Y* with index $j \in J$.

## The Graph Laplacian

In the following, we assume that the components of a digraph are strongly connected. For the simplicity of the presentation, we first consider one strongly connected component separately.

### One Component

We consider a strongly connected, simple, directed graph $G = (V,E)$ with a finite set of vertices $V = \{1, \ldots , m \}$ and a set of edges $E \subseteq V \times V$. Further, we consider positive edge labels $k \in {\mathbb R}^E_>$ and the resulting labeled digraph $G_k=(V,E,k)$. Its Laplacian matrix $A_k \in {\mathbb R}^{V \times V}$ is given by

$$\begin{aligned} A_k = I_E {{\,\textrm{diag}\,}}(k) I_{E,s}^\textsf{T}, \end{aligned}$$

where $I_E \in {\mathbb R}^{V \times E}$ is the incidence matrix and $I_{E,s} \in {\mathbb R}^{V \times E}$ is the “source matrix”. Explicitly,

$$\begin{aligned} (A_k)_{i,j}= & {} {\left\{ \begin{array}{ll} k_{j \rightarrow i}, &{}\quad \text {if } (j \rightarrow i) \in E, \\ -\sum _{(i \rightarrow i') \in E} k_{i \rightarrow i'}, &{}\quad \text {if } i = j, \\ 0, &{}\quad \text {otherwise,} \end{array}\right. } \\ (I_E)_{i,(j \rightarrow j')}= & {} {\left\{ \begin{array}{ll} -1, &{}\quad \text {if } i = j, \\ 1, &{}\quad \text {if } i = j', \\ 0, &{}\quad \text {otherwise,} \end{array}\right. } \end{aligned}$$

and

$$\begin{aligned} (I_{E,s})_{i,(j \rightarrow j')} = {\left\{ \begin{array}{ll} 1, &{}\quad \text {if } i = j, \\ 0, &{}\quad \text {otherwise.} \end{array}\right. } \end{aligned}$$

This definition is used in dynamical systems. For example, *k* is the vector of transition rate constants in the continuous-time, linear process $\frac{\textrm{d} \psi }{\textrm{d} t} = A_k \, \psi$ (with $\psi \in {\mathbb R}^V_\ge$ and $\sum _{i \in V} \psi _i=1$). In other fields, the Laplacian matrix is defined as $A_k^\textsf{T}$, $-A_k$, or $-A_k^\textsf{T}$.

Since *G* is connected, $\ker I_E^\textsf{T}= {{\,\textrm{im}\,}}\bar{1}$, where $\bar{1} \in {\mathbb R}^V$ is the vector with all entries equal to one. Further, $\ker I_{E,s}^\textsf{T}= \{0\}$. Most importantly, since *G* is strongly connected,

3 $$\begin{aligned} \ker A_k = {{\,\textrm{im}\,}}K_k \end{aligned}$$

with a positive vector $K_k \in {\mathbb R}^V_>$ (depending on the rate constants). The entries of $K_k$ (the *tree constants*) can be given explicitly in terms of *k*,

$$\begin{aligned} (K_k)_i = \sum _{(V, E') \in T_i} \; \prod _{(j \rightarrow j') \in E'} k_{j \rightarrow j'}, \quad i \in V, \end{aligned}$$

where $T_i$ is the set of directed spanning trees of *G* rooted at vertex $i \in V$ (and directed towards the root). For a minimal proof of Eq. (3), see (Kandori et al. 1993, Lemma 1) or “Appendix A”. We note that the explicit formula is not crucial for our analysis. Finally, the tree constants $K_k$ correspond to minors of the matrix $-A_k$ which is the content of the matrix-tree theorem (for labeled, directed graphs) (Tutte 1948, Theorem 3.6).

Clearly, the matrix

$$\begin{aligned} - A_k {{\,\textrm{diag}\,}}(K_k) \in {\mathbb R}^{V \times V} \end{aligned}$$

has positive diagonal entries and nonpositive off-diagonal entries. Most importantly, it has zero row and column sums: Indeed, $\bar{1}^\textsf{T}A_k {{\,\textrm{diag}\,}}(K_k) = 0$, and also $A_k {{\,\textrm{diag}\,}}(K_k) \, \bar{1} = A_k \, K_k = 0$. As a consequence, the matrix is diagonally dominant.

The entries of $A_k {{\,\textrm{diag}\,}}(K_k)$ can be given explicitly in terms of *k*. For a derivation of this formula and a discussion of the Birkhoff/von Neumann Theorem (Birkhoff 1946; von Neumann 1953), see “Appendix A”. Again, we note that the explicit formula is not crucial for our analysis.

*Example* Throughout this section, we consider the labeled directed graph $G_k=(V,E,k)$ with $V=\{1,2,3\}$ and $E=\{1 \rightarrow 2, 2 \rightarrow 1, 2 \rightarrow 3, 3 \rightarrow 1\}$, that is,with

$$\begin{aligned} A_k = \begin{pmatrix} -k_{12} &{} k_{21} &{} k_{31} \\ k_{12} &{} -k_{21} -k_{23} &{} 0 \\ 0 &{} k_{23} &{} -k_{31} \end{pmatrix}, \quad K_k = \begin{pmatrix} k_{23} k_{31} + k_{21} k_{31} \\ k_{31} k_{12} \\ k_{12} k_{23} \end{pmatrix}, \end{aligned}$$

and

$$\begin{aligned} A_k {{\,\textrm{diag}\,}}(K_k) = k_{12} k_{23} k_{31} \begin{pmatrix} -1 &{} 0 &{} 1 \\ 1 &{} -1 &{} 0 \\ 0 &{} 1 &{} -1 \end{pmatrix} + k_{12} k_{21} k_{31} \begin{pmatrix} -1 &{} 1 &{} 0 \\ 1 &{} -1 &{} 0 \\ 0 &{} 0 &{} 0 \end{pmatrix}, \end{aligned}$$

see also “Appendix A” for the cycle decomposition of $A_k {{\,\textrm{diag}\,}}(K_k)$. $\blacksquare$

Most importantly, we introduce an *auxiliary* connected directed graph $G_\mathcal {E}= (V,\mathcal {E})$ with the same set of vertices *V* as in $G=(V,E)$, but with an arbitrary set of edges $\mathcal {E}\subseteq V \times V$ such that $|\mathcal {E}| = |V| -1$. That is, $G_\mathcal {E}$ is a directed tree. In particular, it has no cycles. Further, $G_\mathcal {E}$ need not be a subgraph of *G* nor be directed towards a root. The corresponding incidence matrix $I_\mathcal {E}\in {\mathbb R}^{V \times \mathcal {E}}$ is given by

$$\begin{aligned} (I_\mathcal {E})_{i,(j \rightarrow j')} = {\left\{ \begin{array}{ll} -1, &{}\quad \text {if } i = j, \\ 1, &{}\quad \text {if } i = j', \\ 0, &{}\quad \text {otherwise.} \end{array}\right. } \end{aligned}$$

Note that the definitions of the incidence matrices $I_E$ and $I_\mathcal {E}$ agree formally. (Just the sets of edges *E* and $\mathcal {E}$ differ.) Clearly, $\ker I_\mathcal {E}= \{ 0 \}$ and $\ker I_\mathcal {E}^\textsf{T}= {{\,\textrm{im}\,}}\bar{1}$.

#### Proposition 1

Let $G_k=(V,E,k)$ be a strongly connected, labeled, simple digraph and $G_\mathcal {E}= (V,\mathcal {E})$ be an auxiliary digraph. Then, there exists a unique invertible matrix $\mathcal {A}_{k,\mathcal {E}}\in {\mathbb R}^{\mathcal {E}\times \mathcal {E}}$, called the *core matrix* of the graph Laplacian, such that

$$\begin{aligned} A_k {{\,\textrm{diag}\,}}(K_k) = - I_\mathcal {E}\mathcal {A}_{k,\mathcal {E}}I_\mathcal {E}^\textsf{T}. \end{aligned}$$

#### Proof

Since *G* is strongly connected,

$$\begin{aligned} \ker \left( A_k {{\,\textrm{diag}\,}}(K_k) \right) = {{\,\textrm{im}\,}}\bar{1}. \end{aligned}$$

Hence,

$$\begin{aligned} {{\,\textrm{im}\,}}\left( {{\,\textrm{diag}\,}}(K_k) A_k^\textsf{T}\right) = \ker \bar{1}^\textsf{T}= {{\,\textrm{im}\,}}I_\mathcal {E}\end{aligned}$$

and

$$\begin{aligned} {{\,\textrm{diag}\,}}(K_k) A_k^\textsf{T}= I_\mathcal {E}B_{k,\mathcal {E}}^\textsf{T}\end{aligned}$$

for a unique matrix $B_{k,\mathcal {E}} \in {\mathbb R}^{V \times \mathcal {E}}$, where uniqueness follows from $\ker I_\mathcal {E}= \{ 0 \}$. For the same reason, we have

$$\begin{aligned} \ker A_k^\textsf{T}= \ker B_{k,\mathcal {E}}^\textsf{T}\end{aligned}$$

and hence

$$\begin{aligned} {{\,\textrm{im}\,}}B_{k,\mathcal {E}} = {{\,\textrm{im}\,}}A_k. \end{aligned}$$

Since *G* is strongly connected,

$$\begin{aligned} {{\,\textrm{im}\,}}A_k = {{\,\textrm{im}\,}}I_E, \end{aligned}$$

cf. Lemma 12 in “Appendix B”, and further

$$\begin{aligned} {{\,\textrm{im}\,}}I_E = {{\,\textrm{im}\,}}I_\mathcal {E}, \end{aligned}$$

cf. Lemma 13 in “Appendix B”. Altogether, we have

$$\begin{aligned} {{\,\textrm{im}\,}}B_{k,\mathcal {E}} = {{\,\textrm{im}\,}}I_\mathcal {E}\end{aligned}$$

and hence

$$\begin{aligned} B_{k,\mathcal {E}} = - I_\mathcal {E}\mathcal {A}_{k,\mathcal {E}}\end{aligned}$$

for a unique matrix $\mathcal {A}_{k,\mathcal {E}}\in {\mathbb R}^{\mathcal {E}\times \mathcal {E}}$. (The minus sign ensures positive diagonal entries of $\mathcal {A}_{k,\mathcal {E}}$ for particular auxiliary graphs; see below.) Since ${{\,\textrm{rank}\,}}(B_{k,\mathcal {E}})={{\,\textrm{rank}\,}}(I_\mathcal {E})=|\mathcal {E}|$, we have $\ker (\mathcal {A}_{k,\mathcal {E}}) = \ker (B_{k,\mathcal {E}}) = \{0\}$, that is, $\mathcal {A}_{k,\mathcal {E}}$ is invertible. Finally, we obtain

$$\begin{aligned} A_k {{\,\textrm{diag}\,}}(K_k) = B_{k,\mathcal {E}} I_\mathcal {E}^\textsf{T}= - I_\mathcal {E}\mathcal {A}_{k,\mathcal {E}}I_\mathcal {E}^\textsf{T}. \end{aligned}$$

$$\square $$

For an auxiliary digraph $G_\mathcal {E}=(V,\mathcal {E})$, we just required $|\mathcal {E}| = |V| -1$. In the following two results, we assume $G_\mathcal {E}$ to be either of the form $i_1 \rightarrow i_2 \rightarrow \ldots \rightarrow i_m$ (a *chain graph*) or of the form $i_1 \rightarrow i_m$, $i_2 \rightarrow i_m$, ..., $i_{m-1} \rightarrow i_m$ (a *star graph* with root $i_m$).

#### Proposition 2

Let $G_k=(V,E,k)$ be a strongly connected, labeled, simple digraph, and let $G_\mathcal {E}= (V,\mathcal {E})$ be an auxiliary digraph that is a chain graph. Then, $\mathcal {A}_{k,\mathcal {E}}\in {\mathbb R}^{\mathcal {E}\times \mathcal {E}}$, the core matrix of the graph Laplacian, is non-negative with positive diagonal.

#### Proof

Let $G_\mathcal {E}= (V,\mathcal {E})$ be the chain graph

$$\begin{aligned} i_1 \rightarrow i_2 \rightarrow \ldots \rightarrow i_m. \end{aligned}$$

It induces a natural order on the set of vertices *V* (and on the set of edges $\mathcal {E}$). For $i,j \in V$, we write $i \le j$ if $i = j$ or $i \rightarrow \ldots \rightarrow j$. An “inverse” of the incidence matrix $I_\mathcal {E}\in {\mathbb R}^{V \times \mathcal {E}}$ is given by $J_\mathcal {E}\in {\mathbb R}^{\mathcal {E}\times V}$ with

$$\begin{aligned} (J_\mathcal {E})_{(i \rightarrow i'),j} = {\left\{ \begin{array}{ll} 1, &{}\quad \text {if } j \le i, \\ 0, &{}\quad \text {otherwise}. \end{array}\right. } \end{aligned}$$

Explicitly, using the order $i_1, \, i_2, \, \ldots , i_m$ on *V*,

$$\begin{aligned} J_\mathcal {E}= \begin{pmatrix} 1 &{} 0 &{} 0 &{} \cdots &{} 0 &{} 0 \\ 1 &{} 1 &{} 0 &{} \ddots &{} 0 &{} 0 \\ \vdots &{} \vdots &{} \ddots &{} \ddots &{} \vdots &{} \vdots \\ 1 &{} 1 &{} 1 &{} \ddots &{} 0 &{} 0 \\ 1 &{} 1 &{} 1 &{} \cdots &{} 1 &{} 0 \end{pmatrix}, \quad I_\mathcal {E}= \begin{pmatrix} -1 &{} 0 &{} \cdots &{} 0 &{} 0 \\ 1 &{} -1 &{} \ddots &{} \vdots &{} \vdots \\ 0 &{} 1 &{} \ddots &{} 0 &{} \vdots \\ \vdots &{} 0 &{} \ddots &{} -1 &{} 0 \\ \vdots &{} \vdots &{} \ddots &{} 1 &{} -1 \\ 0 &{} 0 &{} \cdots &{} 0 &{} 1 \end{pmatrix}, \end{aligned}$$

and indeed, $J_\mathcal {E}I_\mathcal {E}= - \textrm{I}$, where $\textrm{I} \in {\mathbb R}^{\mathcal {E}\times \mathcal {E}}$ is the identity matrix. That is, $-J_\mathcal {E}$ is a generalized left-inverse of $I_\mathcal {E}$. Hence, by Proposition 1,

$$\begin{aligned} \mathcal {A}_{k,\mathcal {E}}= - J_\mathcal {E}A_k {{\,\textrm{diag}\,}}(K_k) J_\mathcal {E}^\textsf{T}. \end{aligned}$$

For an arbitrary matrix $A \in {\mathbb R}^{V \times V}$, 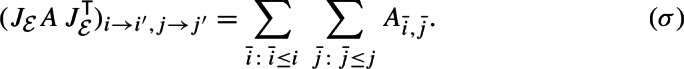 Explicitly, ($\sigma$) is the sum of all entries in the upper left $i \times j$ block of *A*. Now, recall that the matrix $A = -A_k {{\,\textrm{diag}\,}}(K_k)$ has positive diagonal entries and nonpositive off-diagonal as well as zero row and column sums. Hence, the sum ($\sigma$) is nonnegative. Finally, recall that the underlying graph *G* is strongly connected. If $i \rightarrow i'$ equals $j \rightarrow j'$, then the sum ($\sigma$) is positive, since the corresponding subgraph with vertices $\{i_1, i_2, \ldots , i\}$ has incoming and outgoing edges. $\square$

*Example (continued)* In the labeled digraph $G_k=(V,E,k)$ introduced above, there are 3 vertices and hence, 6 possible *chain graphs*. For example, for $\mathcal {E}= \{ 1 \rightarrow 2, 2 \rightarrow 3 \}$ (contained in *E*), we find

$$\begin{aligned} \mathcal {A}_{k,\mathcal {E}}= - J_\mathcal {E}A_k {{\,\textrm{diag}\,}}(K_k) J_\mathcal {E}^\textsf{T}= k_{12} k_{23} k_{31} { \begin{pmatrix} 1 &{} 1 \\ 0 &{} 1 \end{pmatrix}} + k_{12} k_{21} k_{31} \begin{pmatrix} 1 &{} 0 \\ 0 &{} 0 \end{pmatrix}, \end{aligned}$$

whereas for $\mathcal {E}= \{ 1 \rightarrow 3, 3 \rightarrow 2\}$ (both edges not contained in *E*), we find

$$\begin{aligned} \mathcal {A}_{k,\mathcal {E}}= k_{12} k_{23} k_{31} { \begin{pmatrix} 1 &{} 0 \\ 1 &{} 1 \end{pmatrix}} + k_{12} k_{21} k_{31} \begin{pmatrix} 1 &{} 1 \\ 1 &{} 1 \end{pmatrix}. \end{aligned}$$

#### Proposition 3

Let $G_k=(V,E,k)$ be a strongly connected, labeled, simple digraph, and let $G_\mathcal {E}= (V,\mathcal {E})$ be an auxiliary digraph that is a star graph. Then, $\mathcal {A}_{k,\mathcal {E}}\in {\mathbb R}^{\mathcal {E}\times \mathcal {E}}$, the core matrix of the graph Laplacian, is (row and column) diagonally dominant with positive diagonal and non-positive off-diagonal entries.

Explicitly, let $G_\mathcal {E}= (V,\mathcal {E})$ have root $i_m \in V$. Then, $\mathcal {A}_{k,\mathcal {E}}\in {\mathbb R}^{\mathcal {E}\times \mathcal {E}}$ equals $-A_k {{\,\textrm{diag}\,}}(K_k) \in {\mathbb R}^{V \times V}$ with row $i_m$ and column $i_m$ removed and edges ${(i \rightarrow i_m) \in \mathcal {E}}$ identified with vertices $i \in V {\setminus } \{i_m\}$.

#### Proof

Let $G_\mathcal {E}= (V,\mathcal {E})$ be the star graph

$$\begin{aligned} i_1 \rightarrow i_m, \, i_2 \rightarrow i_m, \, \ldots , \, i_{m-1} \rightarrow i_m. \end{aligned}$$

An “inverse” of the incidence matrix $I_\mathcal {E}\in {\mathbb R}^{V \times \mathcal {E}}$ is given by $J_\mathcal {E}\in {\mathbb R}^{\mathcal {E}\times V}$ with

$$\begin{aligned} (J_\mathcal {E})_{(i \rightarrow i'),j} = {\left\{ \begin{array}{ll} 0, &{}\quad \text {if } j=i, \\ 1, &{}\quad \text {otherwise}. \end{array}\right. } \end{aligned}$$

Explicitly, using the order $i_1, \, i_2, \, \ldots , i_m$ on *V*,

$$\begin{aligned} J_\mathcal {E}= \begin{pmatrix} 0 &{} 1 &{} \cdots &{} \cdots &{} 1 &{} 1 \\ 1 &{} 0 &{} 1 &{} \cdots &{} 1 &{} 1 \\ \vdots &{} \ddots &{} \ddots &{} \ddots &{} \vdots &{} \vdots \\ 1 &{} \cdots &{} 1 &{} 0 &{} 1 &{} 1 \\ 1 &{} \cdots &{} \cdots &{} 1 &{} 0 &{} 1 \end{pmatrix}, \quad I_\mathcal {E}= \begin{pmatrix} -1 &{} 0 &{} \cdots &{} 0 &{} 0 \\ 0 &{} -1 &{} \ddots &{} \vdots &{} \vdots \\ \vdots &{} 0 &{} \ddots &{} 0 &{} \vdots \\ \vdots &{} \vdots &{} \ddots &{} 1 &{} 0 \\ 0 &{} 0 &{} \cdots &{} 0 &{} -1 \\ 1 &{} 1 &{} \cdots &{} 1 &{} 1 \end{pmatrix}, \end{aligned}$$

and indeed, $J_\mathcal {E}I_\mathcal {E}= \textrm{I}^{\mathcal {E}\times \mathcal {E}}$. That is, $J_\mathcal {E}$ is a generalized left-inverse of $I_\mathcal {E}$. Hence, by Proposition 1,

$$\begin{aligned} \mathcal {A}_{k,\mathcal {E}}= - J_\mathcal {E}A_k {{\,\textrm{diag}\,}}(K_k) J_\mathcal {E}^\textsf{T}. \end{aligned}$$

For an arbitrary matrix $A \in {\mathbb R}^{V \times V}$, 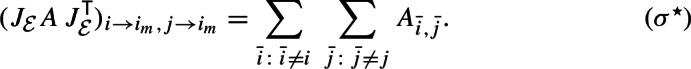 That is, ($\sigma ^\star$) is the sum of all entries of *A* except the entries in row *i* and column *j*. Now, recall that the matrix $A = -A_k {{\,\textrm{diag}\,}}(K_k)$ has zero row and column sums. Hence, ($\sigma ^\star$) equals the sum of all entries (which is zero) *minus* the sums of all entries in row *i* and column *j* (which are zero) *plus* the common entry of row *i* and column *j*. That is,

$$\begin{aligned} (\mathcal {A}_{k,\mathcal {E}})_{i \rightarrow i_m,j \rightarrow i_m} = - (J_\mathcal {E}A_k {{\,\textrm{diag}\,}}(K_k) \, J_\mathcal {E}^\textsf{T})_{i \rightarrow i_m,j \rightarrow i_m} = - (A_k)_{i,j} (K_k)_{j}. \end{aligned}$$

As claimed, $\mathcal {A}_{k,\mathcal {E}}$ equals ${A =} -A_k {{\,\textrm{diag}\,}}(K_k)$ with row $i_m$ and column $i_m$ removed. Like *A*, it has positive diagonal entries and nonpositive off-diagonal entries and is (row and column) diagonally dominant. (However, not all row and column sums are zero.) $\square$

*Example (continued)* In the labeled digraph $G_k=(V,E,k)$ introduced above, there are 3 vertices and hence, 3 possible *star graphs*. For example, for $\mathcal {E}= \{ 2 \rightarrow 1, 3 \rightarrow 1 \}$ (contained in *E*), we find

$$\begin{aligned} \mathcal {A}_{k,\mathcal {E}}= - J_\mathcal {E}A_k {{\,\textrm{diag}\,}}(K_k) J_\mathcal {E}^\textsf{T}= k_{12} k_{23} k_{31} \begin{pmatrix} 1 &{} 0 \\ -1 &{} 1 \end{pmatrix} + k_{12} k_{21} k_{31} \begin{pmatrix} 1 &{} 0 \\ 0 &{} 0 \end{pmatrix}, \end{aligned}$$

whereas for $\mathcal {E}= \{ 1 \rightarrow 3, 2 \rightarrow 3 \}$ (first edge not contained in *E*), we find

$$\begin{aligned} \mathcal {A}_{k,\mathcal {E}}= k_{12} k_{23} k_{31} \begin{pmatrix} 1 &{} 0 \\ -1 &{} 1 \end{pmatrix} + k_{12} k_{21} k_{31} \begin{pmatrix} 1 &{} -1 \\ -1 &{} 1 \end{pmatrix}. \end{aligned}$$

*Remark* In applications to mass-action systems in Sect. 3, we use chain graphs (rather than star graphs).

### Several Components

In general, we consider a labeled, simple digraph $G_k=(V,E,k)$ with $\ell$ strongly connected components $G_k^{\lambda }= ({V^{\lambda }}, {E^{\lambda }}, k^{\lambda })$, $\lambda =1,\ldots ,\ell$, finite sets of vertices ${V^{\lambda }}$, sets of edges ${E^{\lambda }}\subseteq {V^{\lambda }}\times {V^{\lambda }}$, and positive edge labels $k^{\lambda }\in {\mathbb R}^{{E^{\lambda }}}_>$. The corresponding Laplacian matrix $A_k \in {\mathbb R}^{V \times V}$ is block-diagonal with blocks $A_k^{\lambda }\in {\mathbb R}^{{V^{\lambda }}\times {V^{\lambda }}}$, and the vector of tree constants $K_k \in {\mathbb R}^V_>$ has blocks $K_k^{\lambda }\in {\mathbb R}^{{V^{\lambda }}}_>$. Explicitly,

$$\begin{aligned} A_k = \begin{pmatrix} A_k^1 &{} &{} 0 \\ {} &{} \ddots &{} \\ 0 &{} &{} A_k^\ell \end{pmatrix} \in {\mathbb R}^{V \times V} \quad \text {and}\quad K_k = \begin{pmatrix} K_k^1 \\ \vdots \\ K_k^\ell \end{pmatrix} \in {\mathbb R}^V_>. \end{aligned}$$

Accordingly, an auxiliary digraph $G_\mathcal {E}= (V,\mathcal {E})$ has $\ell$ connected components $G_\mathcal {E}^{\lambda }= ({V^{\lambda }}, {\mathcal {E}^{\lambda }})$ with ${\mathcal {E}^{\lambda }}\subseteq {V^{\lambda }}\times {V^{\lambda }}$ and $|{\mathcal {E}^{\lambda }}| = |{V^{\lambda }}| -1$. The corresponding incidence matrix $I_\mathcal {E}\in {\mathbb R}^{V \times \mathcal {E}}$ is block-diagonal with blocks $I_\mathcal {E}^{\lambda }\in {\mathbb R}^{{V^{\lambda }}\times {\mathcal {E}^{\lambda }}}$. We say that $G_\mathcal {E}$ is a chain graph, if each component of $G_\mathcal {E}$ is a chain graph, and analogously for a star graph.

Propositions 1, 2, and 3 imply the main result of this section.

#### Theorem 4

Let $G_k=(V,E,k)$ be a labeled, simple digraph with strongly connected components, and let $G_\mathcal {E}= (V,\mathcal {E})$ be an auxiliary digraph. Then, there exists an invertible, block-diagonal matrix $\mathcal {A}_{k,\mathcal {E}}\in {\mathbb R}^{\mathcal {E}\times \mathcal {E}}$, called the *core matrix* of the graph Laplacian, such that

$$\begin{aligned} A_k {{\,\textrm{diag}\,}}(K_k) = - I_\mathcal {E}\mathcal {A}_{k,\mathcal {E}}I_\mathcal {E}^\textsf{T}. \end{aligned}$$

If $G_\mathcal {E}$ is a chain graph, then $\mathcal {A}_{k,\mathcal {E}}$ is non-negative with positive diagonal. If $G_\mathcal {E}$ is a star graph, then $\mathcal {A}_{k,\mathcal {E}}$ is diagonally dominant with positive diagonal and non-positive off-diagonal entries.

Explicitly,

$$\begin{aligned} I_\mathcal {E}= \begin{pmatrix} I_\mathcal {E}^1 &{} &{} 0 \\ {} &{} \ddots &{} \\ 0 &{} &{} I_\mathcal {E}^\ell \end{pmatrix} \in {\mathbb R}^{V \times \mathcal {E}} \quad \text {and}\quad \mathcal {A}_{k,\mathcal {E}}= \begin{pmatrix} \mathcal {A}_{k,\mathcal {E}}^1 &{} &{} 0 \\ {} &{} \ddots &{} \\ 0 &{} &{} \mathcal {A}_{k,\mathcal {E}}^\ell \end{pmatrix} \in {\mathbb R}^{\mathcal {E}\times \mathcal {E}}. \end{aligned}$$

Note that $|{\mathcal {E}^{\lambda }}| = |{V^{\lambda }}|-1$, $\lambda =1,\ldots ,\ell$, and hence, $|\mathcal {E}|=|V|-\ell$. That is, an auxiliary graph has $|\mathcal {E}|=|V|-\ell$ edges, and a core matrix has $|\mathcal {E}|=|V|-\ell$ rows and columns.

## Mass-Action Systems

We apply the graph-theoretic/algebraic results from the previous section to mass-action systems. We start with a brief summary of fundamental concepts and results.

A *chemical reaction network* (*G*, *y*) is given by a simple directed graph $G = (V,E)$ with a finite set of vertices $V = \{1, \ldots , m \}$ and a set of edges *(reactions)* $E \subseteq V \times V$ together with an injective map $y :V \rightarrow {\mathbb R}^n_\ge$ (a matrix $Y \in {\mathbb R}^{n \times V}_\ge$), assigning to every vertex $i \in V$ a *complex* $y(i) \in {\mathbb R}^n_\ge$. (The digraph *G* is “embedded” in ${\mathbb R}^n_\ge$.) If the components of *G* (the *linkage classes*) are strongly connected, then the network is called *weakly reversible*.

A *mass-action system* $(G_k,y)$ is a chemical reaction network (*G*, *y*) where every edge $(i \rightarrow i') \in E$ is labeled with a *rate constant* $k_{i \rightarrow i'} > 0$, yielding the labeled, simple digraph $G_k = (V,E,k)$ with $k \in {\mathbb R}^E_>$. (If the network is weakly reversible, then also the mass-action system is called weakly reversible.)

The resulting dynamical system for $x \in {\mathbb R}^n_\ge$ (the *concentrations* of *n* molecular species) is given by

$$\begin{aligned} \frac{\textrm{d} x}{\textrm{d} t} = f_k(x) = \sum _{(i \rightarrow i') \in E} k_{i \rightarrow i'} \, x^{y(i)} \left( y(i') - y(i)\right) . \end{aligned}$$

The right-hand side of the ODE can be decomposed as

$$\begin{aligned} f_k(x) = Y I_E {{\,\textrm{diag}\,}}(k) I_{E,s}^\textsf{T}\, x^Y = Y A_k \, x^Y \end{aligned}$$

where $I_E \in {\mathbb R}^{V \times E}$ is the incidence matrix, $I_{E,s} \in {\mathbb R}^{V \times E}$ is the “source matrix”, and

$$\begin{aligned} A_k = I_E {{\,\textrm{diag}\,}}(k) I_{E,s}^\textsf{T}\in {\mathbb R}^{V \times V} \end{aligned}$$

is the resulting Laplacian matrix of the labeled, simple digraph $G_k$. In the following, we consider the dynamical system in the form

4 $$\begin{aligned} \frac{\textrm{d} x}{\textrm{d} t} = f_k(x) = Y A_k \, x^Y. \end{aligned}$$

The *stoichiometric subspace* is given by $S = {{\,\textrm{im}\,}}(Y I_E).$ Clearly, $\frac{\textrm{d} x}{\textrm{d} t} = f_k(x) \in S$, and hence, $x(t) \in x(0)+S$. For $x' \in {\mathbb R}^n_>$, the forward invariant set $(x'+S) \cap {\mathbb R}^n_\ge$ is called a positive *stoichiometric (compatibility) class*.

If an equilibrium $x \in {\mathbb R}^n_>$ of the ODE fulfills

5 $$\begin{aligned} A_k \, x^Y = 0, \end{aligned}$$

then it is a positive *complex-balanced* equilibrium (CBE), also known as vertex-balanced steady state.

*Remark* In the linear setting, the Laplacian matrix captures state transitions on a graph. Let $\psi = x^Y$ be the state variable, given by the vector of monomials. If $A_k \, \psi = 0$, then transitions are balanced (at every vertex of the graph), and *x* is a CBE. If $Y A_k \, x^Y = 0$ (but not $A_k \, x^Y = 0$), then *x* is a general equilibrium.

As shown by Horn (1972) and Horn and Jackson (1972), if there exists a positive CBE (in some stoichiometric class), then

1. the mass-action system is weakly reversible (Horn 1972, Theorem 3C),
2. the equilibrium is asymptotically stable, and all equilibria are complex- balanced (Horn and Jackson 1972, Theorem 6A), and,
3. there exists a unique positive (necessarily complex-balanced) equilibrium in every stoichiometric class (Horn and Jackson 1972, Lemma 4B).

In the following remarks, we elaborate on results 1, 2, and 3.

*Remark (result 1)* Let *G* be weakly reversible and $G_\mathcal {E}= (V,\mathcal {E})$ be some auxiliary digraph. By Theorem 4, $A_k = - I_\mathcal {E}\mathcal {A}_{k,\mathcal {E}}I_\mathcal {E}^\textsf{T}{{\,\textrm{diag}\,}}(K_k^{-1})$. Further, $\ker (I_\mathcal {E}) = \ker (\mathcal {A}_{k,\mathcal {E}}) = \{0\}$. Hence, a positive CBE $x \in {\mathbb R}^n_>$ is given by

$$\begin{aligned} I_\mathcal {E}^\textsf{T}{{\,\textrm{diag}\,}}(K_k^{-1}) \, x^Y = 0, \end{aligned}$$

that is, by the binomial equations

6 $$\begin{aligned} \frac{x^{y(i')}}{(K_k)_{i'}} - \frac{x^{y(i)}}{(K_k)_i} = 0 \quad \text {for } (i \rightarrow i') \in \mathcal {E}. \end{aligned}$$

Given a particular positive CBE $x^* \in {\mathbb R}^n_>$, Eq. (6) is equivalent to

$$\begin{aligned} \left( \frac{x}{x^*}\right) ^{y(i')} = \left( \frac{x}{x^*}\right) ^{y(i)} \quad \text {for } (i \rightarrow i') \in \mathcal {E}\end{aligned}$$

and further to $(y(i')-y(i))^\textsf{T}\ln (x/x^*)=0$ for $(i \rightarrow i') \in \mathcal {E}=0$, that is, to ${(Y I_\mathcal {E})^\textsf{T}\ln (x/x^*) = 0}$. Since $\ker (Y I_\mathcal {E})^\textsf{T}= ({{\,\textrm{im}\,}}Y I_\mathcal {E})^\perp = ({{\,\textrm{im}\,}}Y I_E)^\perp = S^\perp$, the set of all positive CBEs is given by the monomial parametrization $x= x^* \circ {{\,\textrm{e}\,}}^{S^\perp }$.

*Remark (result 2)* In Sect. 3.3, we extend the classical stability result. As it turns out, it holds not only for complex-balanced equilibria of mass-action systems, but for all equilibria of binomial differential inclusions.

In “Appendix C”, we give another proof for the asymptotic stability of complex-balanced equilibria (and the non-existence of other steady states) without using differential inclusions.

*Remark (result 3)* Technically, result 3 states that $|(x^* \circ {{\,\textrm{e}\,}}^{S^\perp }) \cap (x'+S)| = 1$, for all $x^*, x' \in {\mathbb R}^n_>$. An equivalent result appears in toric geometry (Fulton 1993), where it is related to moment maps, and in statistics (Pachter and Sturmfels 2005), where it is related to log-linear models and called Birch’s theorem after (Birch 1963). For generalizations, see Müller and Regensburger (2012), Müller and Regensburger (2014), Müller et al. (2019), Craciun et al. (2019) and Gopalkrishnan et al. (2014).

### Binomial Structure

Given that the network is weakly reversible (the components of the graph *G* are strongly connected), our main graph-theoretic/algebraic result, Theorem 4, implies that the dynamical system (4) for the mass-action system $(G_k,y)$ can be decomposed as

7 $$\begin{aligned} \frac{\textrm{d} x}{\textrm{d} t} = f_{k,\mathcal {E}}(x) = - Y I_\mathcal {E}\mathcal {A}_{k,\mathcal {E}}I_\mathcal {E}^\textsf{T}{{\,\textrm{diag}\,}}(K_k^{-1}) \, x^Y, \end{aligned}$$

where $G_\mathcal {E}= (V,\mathcal {E})$ is some auxiliary digraph.

Again, we have a closer look at the term $I_\mathcal {E}^\textsf{T}{{\,\textrm{diag}\,}}(K_k^{-1}) \, x^Y \in {\mathbb R}^\mathcal {E}$. Indeed,

$$\begin{aligned} \left( I_\mathcal {E}^\textsf{T}{{\,\textrm{diag}\,}}(K_k^{-1}) \, x^Y \right) _{i \rightarrow i'} = \frac{x^{y(i')}}{(K_k)_{i'}} - \frac{x^{y(i)}}{(K_k)_i} \quad \text {for } (i \rightarrow i') \in \mathcal {E}. \end{aligned}$$

That is, the right-hand side of the dynamical system is a sum of binomials. This is obvious for symmetric digraphs (reversible networks); cf. (Craciun et al. 2009, Eq. (14)). By Theorem 4, it also holds for digraphs with strongly connected components (weakly reversible networks).

In particular, for a complex-balanced equilibrium, not just the right-hand side of (7) is zero, but every individual binomial is zero. In this sense, the ODE (7) does not only have binomial *steady states* (positive complex-balanced equilibria, given by binomial equations), but truly is a binomial *dynamical system*.

### Monomial Evaluation Orders and Corresponding Polyhedra/Polyhedral Cones

Let $(G_k,y)$ be a mass-action system based on the labeled, simple digraph $G_k=(V,E,k)$ and the map *y* (the matrix *Y*).

For fixed $x \in {\mathbb R}^n_>$, the values of the monomials $x^{y(i)}$ with $i \in V$ are ordered (using the order on ${\mathbb R}$). For simplicity, we first consider a connected graph $G=(V,E)$. Obviously, the *total* order

$$\begin{aligned} x^{y(i_1)} \le x^{y(i_2)} \le \ldots \le x^{y(i_m)} \end{aligned}$$

can be represented by a chain graph,

$$\begin{aligned} i_1 \rightarrow i_2 \rightarrow \ldots \rightarrow i_m. \end{aligned}$$

If the order is non-strict (if some monomials have the same value), then the representation is not unique. Analogously, the *partial* order

$$\begin{aligned} x^{y(i_1)} \le x^{y(i_m)}, \, x^{y(i_2)} \le x^{y(i_m)}, \, \ldots , \, x^{y(i_{m-1})} \le x^{y(i_m)} \end{aligned}$$

can be represented by a star graph,

$$\begin{aligned} i_1 \rightarrow i_m, \, i_2 \rightarrow i_m, \, \ldots , \, i_{m-1} \rightarrow i_m. \end{aligned}$$

In general, every auxiliary graph $G_\mathcal {E}=(V,\mathcal {E})$ represents a partial order on the vertices of *G* and hence, on the values of the monomials.

In the following, we will consider monomials with coefficients:

- $\frac{x^{y(i)}}{(K_k)_i}$, for weakly reversible networks with tree constants $K_k \in {\mathbb R}^V_>$, and
- $(\frac{x}{x^*})^{y(i)}$, for given positive CBE $x^* \in {\mathbb R}^n_>$.

#### Weak Reversibility

Let $(G_k,y)$ be a weakly reversible mass-action system, and fix $x \in {\mathbb R}^n_>$.

We call an order on the entries of $\frac{x^Y}{K_k} \in {\mathbb R}^V_>$ that is total within connected components, but does not relate entries in different components, a *monomial evaluation order* (since the notion *monomial order(-ing)* has a different meaning in algebra). We represent the order by a chain graph $G_{\mathcal {E}} = (V,\mathcal {E})$ and often just by the set of edges $\mathcal {E}$. Explicitly, $(i \rightarrow i') \in \mathcal {E}$ implies $\frac{x^{y(i)}}{(K_k)_i} \le \frac{x^{y(i')}}{(K_k)_{i'}}$. Thereby, the vertices $i,i' \in V$ are necessarily in the same component. If the order is non-strict, then $\mathcal {E}$ is not unique.

Analogously, the maximal entries of $\frac{x^Y}{K_k} \in {\mathbb R}^V_>$ within connected components are greater or equal than all other entries in the respective components. We represent this order by a star graph $G_{\mathcal {E}} = (V,\mathcal {E})$. If there is more than one maximal entry within a component, then $\mathcal {E}$ is not unique.

Conversely, fix an auxiliary graph $G_\mathcal {E}=(V,\mathcal {E})$, for example, a chain graph or a star graph. The subset of ${\mathbb R}^n_>$ with monomial evaluation order represented by $\mathcal {E}$ is given by

8 $$\begin{aligned} \begin{aligned} \mathcal {S}_{k,\mathcal {E}}&= \left\{ x \in {\mathbb R}^n_> \mid \frac{x^{y(i')}}{(K_k)_{i'}} - \frac{x^{y(i)}}{(K_k)_i} \ge 0 \text { for } (i \rightarrow i') \in \mathcal {E}\right\} \\&= \left\{ x \in {\mathbb R}^n_> \mid I_\mathcal {E}^\textsf{T}{{\,\textrm{diag}\,}}(K_k^{-1}) \, x^Y \ge 0 \right\} . \end{aligned} \end{aligned}$$

By the monotonicity of the logarithm,

$$\begin{aligned} \mathcal {S}_{k,\mathcal {E}}&= \left\{ x \in {\mathbb R}^n_> \mid (y(i')-y(i))^\textsf{T}\ln x \ge \ln \frac{(K_k)_{i'}}{(K_k)_i} \text { for } (i \rightarrow i') \in \mathcal {E}\right\} \\&= \left\{ x \in {\mathbb R}^n_> \mid (Y I_\mathcal {E})^\textsf{T}\ln x \ge I_\mathcal {E}^\textsf{T}\ln K_k \right\} . \end{aligned}$$

Hence,

$$\begin{aligned} x \in \mathcal {S}_{k,\mathcal {E}}\quad \Leftrightarrow \quad \ln x \in \mathcal {P}_{k,\mathcal {E}}\end{aligned}$$

with the polyhedron

9 $$\begin{aligned} \begin{aligned} \mathcal {P}_{k,\mathcal {E}}&= \left\{ z \in {\mathbb R}^n \mid (Y I_\mathcal {E})^\textsf{T}z \ge I_\mathcal {E}^\textsf{T}\ln K_k \right\} . \end{aligned} \end{aligned}$$

#### Complex Balancing

If there exists a positive CBE $x^*\in {\mathbb R}^n_>$, then the polyhedra become polyhedral cones.

Fix an auxiliary graph $G_\mathcal {E}=(V,\mathcal {E})$. Using complex balancing (6) for $x^*$, the subset (8) can be written as

$$\begin{aligned} \mathcal {S}_{k,\mathcal {E}}&= \left\{ x \in {\mathbb R}^n_> \mid \left( \frac{x}{x^*}\right) ^{y(i')} - \left( \frac{x}{x^*}\right) ^{y(i)} \ge 0 \text { for } (i \rightarrow i') \in \mathcal {E}\right\} \\&= \left\{ x \in {\mathbb R}^n_> \mid I_\mathcal {E}^\textsf{T}\left( \frac{x}{x^*}\right) ^Y \ge 0 \right\} . \end{aligned}$$

By the monotonicity of the logarithm,

$$\begin{aligned} \mathcal {S}_{k,\mathcal {E}}&= \left\{ x \in {\mathbb R}^n_> \mid (y(i')-y(i))^\textsf{T}\ln \frac{x}{x^*} \ge 0 \text { for } (i \rightarrow i') \in \mathcal {E}\right\} \\&= \left\{ x \in {\mathbb R}^n_> \mid (Y I_\mathcal {E})^\textsf{T}\ln \frac{x}{x^*} \ge 0 \right\} . \nonumber \end{aligned}$$

Hence,

$$\begin{aligned} x \in \mathcal {S}_{k,\mathcal {E}}\quad \Leftrightarrow \quad \ln \frac{x}{x^*} \in \mathcal {C}_\mathcal {E}\end{aligned}$$

with the polyhedral cone

10 $$\begin{aligned} \mathcal {C}_\mathcal {E}&= \left\{ z \in {\mathbb R}^n \mid (Y I_\mathcal {E})^\textsf{T}z \ge 0 \right\} , \end{aligned}$$

which does not depend on *k*. (Of course, $x^*$ depends on *k*.) The lineality space of $\mathcal {C}_\mathcal {E}$ does not even depend on $\mathcal {E}$,

$$\begin{aligned} {{\,\textrm{lineal}\,}}\mathcal {C}_\mathcal {E}= \ker \, (Y I_\mathcal {E})^\textsf{T}= ({{\,\textrm{im}\,}}Y I_\mathcal {E})^\perp = ({{\,\textrm{im}\,}}Y I_E)^\perp = S^\perp . \end{aligned}$$

Obviously, $S^\perp = {{\,\textrm{lineal}\,}}\mathcal {C}_\mathcal {E}\subseteq \mathcal {C}_\mathcal {E}$. For fixed $\mathcal {E}$, there are two possibilities:

- $\mathcal {C}_\mathcal {E}= S^\perp$. Then, all defining (non-strict) inequalities of $\mathcal {C}_\mathcal {E}$ (and $\mathcal {S}_{k,\mathcal {E}}$) are fulfilled with equality, and $\mathcal {S}_{k,\mathcal {E}}= x^* \circ {{\,\textrm{e}\,}}^{S^\perp }$ equals the set of complex-balanced equilibria.
- $\mathcal {C}_\mathcal {E}\supset S^\perp$. Then, $\mathcal {C}_\mathcal {E}$ and $\mathcal {S}_{k,\mathcal {E}}$ are full-dimensional, and the monomial evaluation order is strict in the interior of $\mathcal {S}_{k,\mathcal {E}}$ and non-strict on the boundary (where some monomials have the same value).

In the following study of complex-balanced mass-action systems (and their extension to binomial differential inclusions), we use chain graphs $G_\mathcal {E}$, representing monomial evaluation orders. In this setting, a full-dimensional subset $\mathcal {S}_{k,\mathcal {E}}$ is called a *stratum*, cf. Siegel and Johnston (2011). This term has also been used for partial orders related to the original graph, rather than to an auxiliary graph, cf. Craciun et al. (2009).

*Remark* As stated above, for every $x \in {\mathbb R}^n_>$, there is a (non-unique) $\mathcal {E}$ such that $x \in \mathcal {S}_{k,\mathcal {E}}$. In particular, ${\mathbb R}^n_>$ is a union of strata which intersect only on their boundaries. Correspondingly, ${\mathbb R}^n$ is a union of polyhedral cones $\mathcal {C}_\mathcal {E}$. Indeed, by the monotonicity of the logarithm, an order on the entries of ${(\frac{x}{x^*})^Y \in {\mathbb R}^V_>}$ (within components) is equivalent to an order on the entries of $Y^\textsf{T}z \in {\mathbb R}^V$ with $z = \ln \frac{x}{x^*}$, and the set of pairs of vertices within components,

$$\begin{aligned} \Omega = \left\{ i \rightarrow i' \mid i,i' \in V^\lambda , \, \lambda =1,\ldots ,\ell \right\} , \end{aligned}$$

induces an arrangement of central hyperplanes,

$$\begin{aligned} h_{i \rightarrow i'} = \{ z \in {\mathbb R}^n \mid ( y(i') - y(i))^T z = 0 \}, \quad (i \rightarrow i') \in \Omega . \end{aligned}$$

The central hyperplane arrangement decomposes ${\mathbb R}^n$ into open polyhedral cones called *faces*; full dimensional faces are called *cells*. In our terminology, a cell is the interior of a polyhedral cone $\mathcal {C}_\mathcal {E}$ and hence, corresponds to the interior of a stratum $\mathcal {S}_{k,\mathcal {E}}$.

*Example* Let $(G_k,y)$ be a mass-action system given by a strongly connected graph $G=(V,E)$ with $V=\{1,2,3\}$ (and arbitrary *E*) and $y(1) = {2 \atopwithdelims ()1}$, $y(2) = {0 \atopwithdelims ()2}$, $y(3) = {1 \atopwithdelims ()0}$. For simplicity, assume $x^* = {1 \atopwithdelims ()1}$. The corresponding monomials are $(x/x^*)^{y(1)} = x^{y(1)} = x_1^2 x_2$, $x^{y(2)} = x_2^2$, and $x^{y(3)} = x_1$.
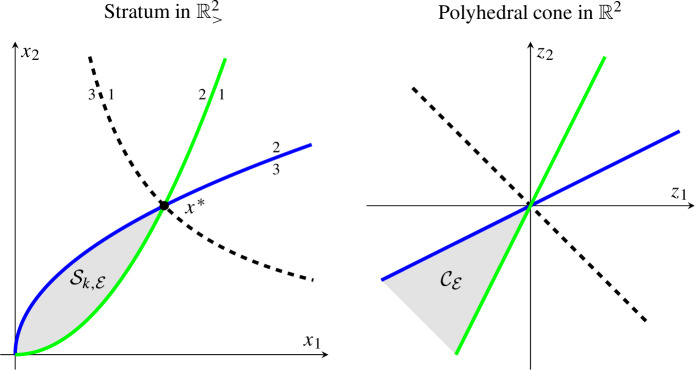
 The positive orthant is a union of strata corresponding to monomial evaluation orders. In particular, consider the stratum given by the order $x^{y(1)} \le x^{y(2)} \le x^{y(3)}$, that is, $\mathcal {S}_{k,\mathcal {E}}$ with $\mathcal {E}= \{1 \rightarrow 2, 2 \rightarrow 3\}$, bounded by the green and blue lines. The green line specifies $x^{y(1)} = x^{y(2)}$; above it, $x^{y(2)} > x^{y(1)}$, as indicated by the corresponding vertices 2 and 1. The blue line specifies $x^{y(2)} = x^{y(3)}$; below it, $x^{y(3)} > x^{y(2)}$. (The dashed black line specifies $x^{y(1)} = x^{y(3)}$, which does not bound the particular stratum.) In the interior of $\mathcal {S}_{k,\mathcal {E}}$, the order is strict. In logarithmic coordinates $z = \ln (x/x^*)$, the stratum corresponds to the polyhedral cone $\mathcal {C}_\mathcal {E}$.

Finally, let *G* have two strongly connected components $G'=(V',E')$, $G''=(V'',E'')$ with $V'=\{1,2,3\}$, $V''=\{4,5\}$, *y*(1), *y*(2), *y*(3) as above, and $y(4) = {0 \atopwithdelims ()0}$, $y(5) = {1 \atopwithdelims ()1}$. (Assume $x^* = {1 \atopwithdelims ()1}$, and hence, $(x/x^*)^{y(4)} = x^{y(4)} = 1$, $x^{y(5)} = x_1 x_2$). Consider the order $x^{y(1)} \le x^{y(2)} \le x^{y(3)}$ and $x^{y(4)} \le x^{y(5)}$, that is, $\mathcal {S}_{k,\mathcal {E}}$ with $\mathcal {E}= \{{1 \rightarrow 2}, {2 \rightarrow 3}, {4 \rightarrow 5}\}$. Explicitly, $\mathcal {S}_{k,\mathcal {E}}= \mathcal {S}' \cap \mathcal {S}''$ with $\mathcal {S}' = \{ x \mid x^{y(1)} \le x^{y(2)} \le x^{y(3)} \}$ (as above) and $\mathcal {S}'' = \{ x \mid x^{y(4)} \le x^{y(5)} \}$ (the region on and above the red line). As a consequence, $\mathcal {S}_{k,\mathcal {E}}= \{ x^* \}$ is trivial (equals the set of complex-balanced equilibria). In logarithmic coordinates, the corresponding polyhedral cone $\mathcal {C}_\mathcal {E}= \{0\}$ is trivial.
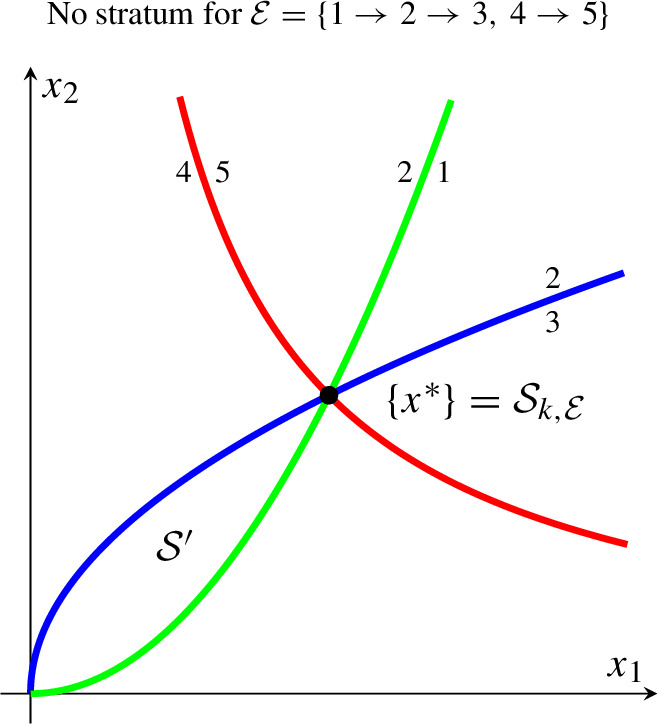


In general, $\mathcal {S}_{k,\mathcal {E}}= x^* \circ {{\,\textrm{e}\,}}^{S^\perp }$ (equals the set of complex-balanced equilibria) if and only if $\mathcal {C}_\mathcal {E}= S^\perp$. In the example, $S = {\mathbb R}^2$ and $S^\perp = \{0\}$.

### Binomial Differential Inclusions

Finally, we extend a classical result by Horn and Jackson from 1972.

#### Theorem 5

(cf. Horn and Jackson 1972, Theorem 6A) Let $(G_k,y)$ be a mass-action system and $x^* \in {\mathbb R}^n_>$ be a positive CBE of the dynamical system (4). Then,

$$\begin{aligned} \left( \ln \frac{x}{x^*} \right) ^\textsf{T}\! f_k(x) < 0 \end{aligned}$$

for all $x \in {\mathbb R}^n_>$ that are not complex-balanced equilibria. Hence, (i) all positive equilibria are complex-balanced, and (ii) $x^*$ is asymptotically stable.

All proofs are based on the entropy-like Lyapunov function $L :{\mathbb R}^n_> \rightarrow {\mathbb R}$,

11 $$\begin{aligned} L(x) = \sum _{i=1}^n x_i \left( \ln \frac{x_i}{x^*_i} -1 \right) + x^*_i. \end{aligned}$$

For $$x \in {\mathbb R}^n_>$$,

$$\begin{aligned} L(x) \ge 0 \quad \text { with ``='' if and only if } x = x^*, \end{aligned}$$

$$\nabla L = \left( \ln \frac{x}{x^*} \right) ^\textsf{T}$$, and hence,

$$\begin{aligned} \frac{\textrm{d} }{\textrm{d} t} \, L(x(t)) = \nabla L \, \frac{\textrm{d} x}{\textrm{d} t} = \left( \ln \frac{x}{x^*} \right) ^\textsf{T}\! f_k(x). \end{aligned}$$

If $\left( \ln \frac{x}{x^*} \right) ^\textsf{T}\! f_k(x) \le 0$ with “=” if and only if $x = x^*$, then *L*(*x*) is a strict Lyapunov function, and $x^*$ is asymptotically stable.

Previous proofs further use inequalities for the exponential function or the logarithm and cycle decomposition of the graph, cf. Horn and Jackson (1972), Sontag (2001), Anderson (2011) and Gopalkrishnan (2014). For a new proof using monomial evaluation orders and corresponding geometric objects (strata and polyhedral cones), see “Appendix C”.

In the following, we extend the stability result and provide a maximally transparent, polyhedral-geometry proof. First, we relate the dynamics in a given stratum to the corresponding polyhedral cone.

In Proposition 6 below, we use the concept of the polar cone

$$\begin{aligned}C^\textrm{pol}= \{ y \mid y \cdot x \le 0 \text { for all } x \in C\}\end{aligned}$$

of a set *C*, where $C^\textrm{pol}\subset ({{\,\textrm{lineal}\,}}C)^\perp$, and $y \in {{\,\textrm{int}\,}}C^\textrm{pol}$ if and only if $y \cdot x < 0$ for all $x \in C {\setminus } {{\,\textrm{lineal}\,}}C$. In our setting, a monomial evaluation order (represented by a chain graph $G_\mathcal {E}$) determines a stratum $\mathcal {S}_{k,\mathcal {E}}$ and a corresponding polyhedral cone $\mathcal {C}_\mathcal {E}$ (which are both full-dimensional). In particular, $\mathcal {C}_\mathcal {E}$ has a non-trivial lineality space ${{\,\textrm{lineal}\,}}\mathcal {C}_\mathcal {E}= S^\perp$ if and only if $S \ne {\mathbb R}^n$, and $\mathcal {C}_\mathcal {E}^\textrm{pol}\subset S$. By Proposition 6, if $\ln \frac{x}{x^*} \in \mathcal {C}_\mathcal {E}$, then $f_k(x) \in \mathcal {C}_\mathcal {E}^\textrm{pol}$.
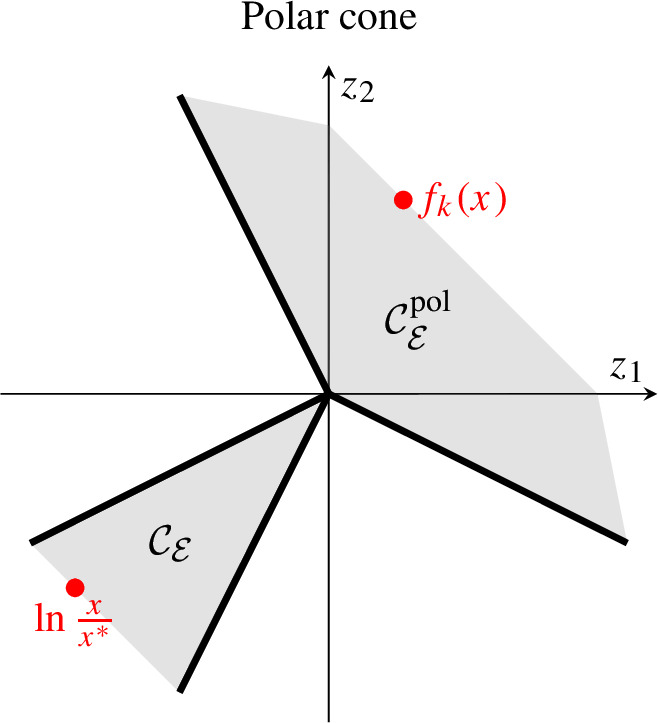


#### Proposition 6

Let $(G_k,y)$ be a complex-balanced mass-action system, $G_\mathcal {E}$ be a chain graph, and $\mathcal {S}_{k,\mathcal {E}}\subset {\mathbb R}^n_>$ be a stratum. Then, for all $x \in \mathcal {S}_{k,\mathcal {E}}$ that are not positive complex-balanced equilibria, $f_k(x) \in {{\,\textrm{int}\,}}\mathcal {C}_\mathcal {E}^\textrm{pol}$.

#### Proof

Let $x \in \mathcal {S}_{k,\mathcal {E}}$ and $u \in \mathcal {C}_\mathcal {E}$. Using the dynamical system (4) and Theorem 4, we have

$$\begin{aligned} u^\textsf{T}\! f_k(x)&= u^\textsf{T}Y A_k \, x^Y \\&= - u^\textsf{T}Y I_\mathcal {E}\mathcal {A}_{k,\mathcal {E}}I_\mathcal {E}^\textsf{T}{{\,\textrm{diag}\,}}(K_k^{-1}) \, x^Y \\&= - \, a^\textsf{T}\mathcal {A}_{k,\mathcal {E}}\, b \end{aligned}$$

with

$$\begin{aligned} a(u)&= (Y I_\mathcal {E})^\textsf{T}u , \\ b(x)&= I_\mathcal {E}^\textsf{T}{{\,\textrm{diag}\,}}(K_k^{-1}) \, x^Y . \end{aligned}$$

Using $\mathcal {S}_{k,\mathcal {E}}$ and $\mathcal {C}_\mathcal {E}$ as in Eqs. (8) and (10), we have $b \ge 0$ and $a \ge 0$.

By Theorem 4, the core matrix of the graph Laplacian, $\mathcal {A}_{k,\mathcal {E}}\in {\mathbb R}^{\mathcal {E}\times \mathcal {E}}$, is non-negative with positive diagonal. Hence,

$$\begin{aligned} u^\textsf{T}\! f_k(x) = - a^\textsf{T}\mathcal {A}_{k,\mathcal {E}}\, b \le 0 \end{aligned}$$

and

$$\begin{aligned} f_k(x) \in \mathcal {C}_\mathcal {E}^\textrm{pol}. \end{aligned}$$

Recall $f_k(x) \in S$. Hence, $u^\textsf{T}\! f_k(x) = 0$ for $u \in {{\,\textrm{lineal}\,}}\mathcal {C}_\mathcal {E}= S^\perp$. So, let $x \in \mathcal {S}_{k,\mathcal {E}}$ not be a CBE, that is, $f_k(x) \ne 0$, and $u \in \mathcal {C}_\mathcal {E}$ not lie in the lineality space, that is, $u \not \in S^\perp$. Then, $u^\textsf{T}\! f_k(x) \ne 0$. Altogether, $u^\textsf{T}\! f_k(x) < 0$ and $f_k(x) \in {{\,\textrm{int}\,}}\mathcal {C}_\mathcal {E}^\textrm{pol}$. $\square$

Now, let $(G_k,y)$ be a mass-action system and $x^* \in {\mathbb R}^n_>$ be a positive CBE of the dynamical system (4). Proposition 6 suggests to introduce a corresponding piece-wise constant *binomial differential inclusion* as

$$\begin{aligned} \frac{\textrm{d} x}{\textrm{d} t} \in F_{x^*}(x) = {\left\{ \begin{array}{ll} \{0\} &{}\quad \text { for } x \in x^* \circ {{\,\textrm{e}\,}}^{S^\perp }, \\ {{\,\textrm{int}\,}}\left( \bigcap _{\mathcal {E}:x \in \mathcal {S}_{k,\mathcal {E}}} \mathcal {C}_\mathcal {E}^\textrm{pol}\right) &{}\quad \text { for } x \not \in x^* \circ {{\,\textrm{e}\,}}^{S^\perp }, \end{array}\right. } \end{aligned}$$

thereby explicitly specifying the set of positive equilibria $x^* \circ {{\,\textrm{e}\,}}^{S^\perp }$. Equivalently, using $x \in \mathcal {S}_{k,\mathcal {E}}$ $\Leftrightarrow$ $\ln \frac{x}{x^*} \in \mathcal {C}_\mathcal {E}$,

12 $$\begin{aligned} \frac{\textrm{d} x}{\textrm{d} t} \in F( \textstyle \ln \frac{x}{x^*}) \quad \text {with} \quad F(u) = {\left\{ \begin{array}{ll} \{0\} &{}\quad \text { for } u \in S^\perp , \\ {{\,\textrm{int}\,}}\left( \bigcap _{\mathcal {E}:u \in \mathcal {C}_\mathcal {E}} \mathcal {C}_\mathcal {E}^\textrm{pol}\right) &{}\quad \text { for } u \not \in S^\perp . \end{array}\right. } \end{aligned}$$

Proposition 6 immediately implies the following result.

#### Theorem 7

Let $(G_k,y)$ be a mass-action system and $x^* \in {\mathbb R}^n_>$ be a positive CBE of the dynamical system (4). Then, the mass-action system can be embedded in the binomial differential inclusion (12).

Finally, we extend Theorem 5 (from complex-balanced mass-action systems to binomial differential inclusions).

#### Theorem 8

Let $x^* \in {\mathbb R}^n_>$ be a positive equilibrium of the binomial differential inclusion (12). Then,

$$\begin{aligned} \left( \ln \frac{x}{x^*} \right) ^\textsf{T}\! f < 0, \end{aligned}$$

for all $x \in {\mathbb R}^n_>$ that are not positive equilibria and all $f \in F(\ln \frac{x}{x^*})$. Hence, $x^*$ is asymptotically stable.

#### Proof

Let $\mathcal {S}_{k,\mathcal {E}}\subset {\mathbb R}^n_>$ be a stratum and $x \in \mathcal {S}_{k,\mathcal {E}}$ not be a positive equilibrium. On the one hand,

$$\begin{aligned} x \in \mathcal {S}_{k,\mathcal {E}}\setminus (x^* \circ {{\,\textrm{e}\,}}^{S^\perp }), \quad \text {that is,} \quad \ln \frac{x}{x^*} \in \mathcal {C}_\mathcal {E}\setminus S^\perp . \end{aligned}$$

that is, $\ln \frac{x}{x^*}$ lies in $\mathcal {C}_\mathcal {E}$, but not in the lineality space ${{\,\textrm{lineal}\,}}\mathcal {C}_\mathcal {E}= S^\perp$. On the other hand,

$$\begin{aligned} f \in F\left( \ln \frac{x}{x^*}\right) = {{\,\textrm{int}\,}}\left( \bigcap _{\mathcal {E}:\ln \! \frac{x}{x^*} \in \mathcal {C}_\mathcal {E}} \mathcal {C}_\mathcal {E}^\textrm{pol}\right) , \end{aligned}$$

and $\mathcal {C}_\mathcal {E}^\textrm{pol}\subset S$. Hence, $\left( \ln \frac{x}{x^*} \right) ^\textsf{T}\! f < 0$, and *L*(*x*) is a strict Lyapunov function. $\square$

#### Remark 9

Even if a weakly reversible mass-action system $(G_k,y)$ does not admit a complex-balanced equilibrium $x^*$, it can be embedded in a piece-wise constant differential inclusion. Technically, the absence of a CBE $x^*$ does not allow to pass from the polyhedron $\mathcal {P}_{k,\mathcal {E}}$ (with given monomial evaluation order) to the cone $\mathcal {C}_\mathcal {E}$, cf. Eqs. (9) and (10). That is, instead of a central hyperplane arrangement (that defines the cones $\mathcal {C}_\mathcal {E}$), one considers a non-central hyperplane arrangement (that defines the polyhedra $\mathcal {P}_{k,\mathcal {E}}$). In analogy to Proposition 6, one can show that, for a chain graph $G_\mathcal {E}$ and a stratum $\mathcal {S}_{k,\mathcal {E}}$, it holds that $f_{k(x)} \in {{\,\textrm{int}\,}}( {{\,\textrm{rec}\,}}( \mathcal {P}_{k,\mathcal {E}})^{\textrm{pol}})$, for all $x \in \mathcal {S}_{k,\mathcal {E}}$. Here, ${{\,\textrm{rec}\,}}(C)$ denotes the *recession cone* of a set *C*.

### Discussion

As Horn and Jackson in 1972 (Horn and Jackson 1972, Theorem 6A), we have shown that, in mass-action systems with a positive complex-balanced equilibrium, every positive equilibrium is complex-balanced and asymptotically stable. For a proof using the new decomposition of the graph Laplacian, monomial evaluation orders, and corresponding geometric objects (strata and polyhedral cones), see “Appendix C”. In fact, we have extended the result to *binomial differential inclusions* (BDIs), introduced in this work. Every positive equilibrium of a BDI is asymptotically stable, see Theorem 8.

#### Binomial and Toric Differential Inclusions

Given a reaction network (*G*, *y*) with graph $G=(V,E)$ and “complex” map $y :V \rightarrow {\mathbb R}^n_\ge$, a BDI depends on the components of the graph (but not on the exact edge set *E*) and on some positive equilibrium $x^*$ (but not explicitly on the rate constants). In fact, it is mainly determined by stoichiometry, namely by pairwise differences of complexes, defining a hyperplane arrangement. In particular, monomial evaluation orders correspond to polyhedral cones (in logarithmic coordinates) and strata (in the original positive variables). More formally, a BDI is given by a hyperplane arrangement (with lineality space $S^\perp$) and a positive equilibrium $x^*$, see Equation (12). Most importantly, complex-balanced mass-action systems can be embedded in BDIs.

Recently, *toric differential inclusions* (TDIs) have been used in a proposed proof (Craciun 2015, 2019) of the global attractor conjecture (Horn 1974), stating that complex-balanced equilibria are not just asymptotically, but also globally stable. In fact, TDIs also allow to tackle the persistence and permanence conjectures for (weakly reversible) mass-action systems with (time-)variable rate constants. In the classical setting, rate constants $k>0$ are fixed, whereas, in the study of the conjectures mentioned above, rate constants $\epsilon \le k(t) \le 1/\epsilon$ may vary over time, but are bounded (Anderson 2011; Craciun et al. 2013). To address this complication, “uncertainty regions” with thickness $\delta (\epsilon )$ around the boundaries of “regions with definite monomial order” are introduced. On the one hand, BDIs are special cases of TDIs with $\delta \rightarrow 0$ (modulo a translation of the hyperplane arrangement by $\log x^*$), and also the piece-wise constant differential inclusions mentioned in Remark 9 can be embedded in TDIs (with $\delta >0$). On the other hand, BDIs allow to consider (the asymptotic stability of) positive equilibria, whereas TDIs capture the dynamics close to the boundary of the positive orthant without being explicit about equilibria.

#### Generalized Mass-Action Systems

In previous work, we have studied *generalized* mass-action systems (Müller and Regensburger 2012, 2014; Müller et al. 2016, 2019; Craciun et al. 2019; Boros et al. 2020). In order to motivate the setting, we consider the reaction ${1 \textsf{X}_1+ 1 \textsf{X}_2 \rightarrow \textsf{X}_3}$ with “stoichiometric” coefficients equal to 1. Under the assumption of generalized mass-action kinetics, its rate is given by $v = k \, (x_1)^a (x_2)^b$ with arbitrary “kinetic orders” $a,b > 0$ (in particular, different from 1). Using the complexes $y = (1,1,0,0,\ldots )^\textsf{T}$, $y'=(0,0,1,0,\ldots )^\textsf{T}$, and the kinetic-order complex $\tilde{y} = (a,b,0,0, \ldots )^\textsf{T}$, we can write the reaction as $y \rightarrow y'$ with rate $v = k \, x^{\tilde{y}}$. For a network, the resulting dynamical system,

13 $$\begin{aligned} \frac{\textrm{d} x}{\textrm{d} t} = Y A_k \, x^{\tilde{Y}}, \end{aligned}$$

is determined by the matrices *Y* (by stoichiometry), $\tilde{Y}$ (by kinetics), and $A_k$ (by a graph). For generalized mass-action systems, asymptotic stability of complex-balanced equilibria and non-existence of other steady states are not guaranteed (as for classical mass-action systems, cf. Theorem 5). We have already provided necessary conditions for linear stability of complex-balanced equilibria (Boros et al. 2020). In parallel work (Müller 2022), we use the new decomposition of the graph Laplacian and monomial evaluation orders to study sufficient conditions for linear stability of complex-balanced equilibria and non-existence of other steady states.

## Data Availability

Data sharing is not applicable to this article as no datasets were generated or analyzed during the current study.

## A Explicit Formulas for $K_k$ and $A_k Diag(K_k)$

We consider a strongly connected, labeled, simple digraph $G_k=(V,E,k)$. Based on the underlying unlabeled graph $G=(V,E)$, we introduce three sets of subgraphs.

1. For $i \in V$, we introduce the set $T_i$ of subgraphs of *G* that fulfill two requirements: (i) a subgraph does not contain a cycle, and (ii) every vertex except *i* is the source of exactly one edge. (This is the set of directed spanning trees of *G* rooted at vertex *i* and directed towards the root).
2. For $i \in V$, we introduce the set $G_i$ of subgraphs of *G* that fulfill three requirements: (i) a subgraph contains exactly one cycle, (ii) this cycle contains vertex *i*, and (iii) every vertex is the source of exactly one edge. (This set has been used in Kandori et al. 1993, Lemma 1).
3. For a cycle *C* contained in *G*, we introduce the set $G_C$ of subgraphs of *G* that fulfill three requirements: (i) a subgraph contains cycle *C*, (ii) *C* is the only cycle, and (iii) every vertex is the source of exactly one edge. (This is the obvious extension from fixing a vertex to fixing a cycle).

In the following, we write $(i \rightarrow i') \in G$ short for $(i \rightarrow i') \in E$, where $G=(V,E)$.

### Fact 10

$$\begin{aligned} A_k K_k = 0, \end{aligned}$$

where

$$\begin{aligned} (K_k)_i = \sum _{T \in T_i} \; \prod _{(j \rightarrow j') \in T} k_{j \rightarrow j'}, \quad \text {for } i \in V. \end{aligned}$$

### Proof

Recall

$$\begin{aligned} (A_k \, \psi )_i = \sum _{(i' \rightarrow i) \in G} k_{i' \rightarrow i} \, \psi _{i'} - \sum _{(i \rightarrow i') \in G} k_{i \rightarrow i'} \, \psi _i \quad \text {for } i \in V. \end{aligned}$$

On the one hand, every subgraph $S \in G_i$ gives rise to a spanning tree $T \in T_i$ and vice versa (by removing/adding the edge $i \rightarrow i'$). For $i \in V$,

$$\begin{aligned} \sum _{S \in G_i} \prod _{(j \rightarrow j') \in S} k_{j \rightarrow j'}&= \sum _{T \in T_i} \sum _{(i \rightarrow i') \in G} \prod _{(j \rightarrow j') \in T} k_{j \rightarrow j'} \cdot k_{i \rightarrow i'} \\&= \sum _{(i \rightarrow i') \in G} k_{i \rightarrow i'} \sum _{T \in T_i} \prod _{(j \rightarrow j') \in T} k_{j \rightarrow j'} \\&= \sum _{(i \rightarrow i') \in G} k_{i \rightarrow i'} \, (K_k)_i . \end{aligned}$$

On the other hand, every subgraph $S \in G_i$ gives rise to a spanning tree $T \in T_{i'}$ and vice versa (by removing/adding the edge $i' \rightarrow i$ that is in the cycle). For $i \in V$,

$$\begin{aligned} \sum _{S \in G_i} \prod _{(j \rightarrow j') \in S} k_{j \rightarrow j'}&= \sum _{T \in T_{i'}} \sum _{(i' \rightarrow i) \in G} \prod _{(j \rightarrow j') \in T} k_{j \rightarrow j'} \cdot k_{i' \rightarrow i} \\&= \sum _{(i' \rightarrow i) \in G} k_{i' \rightarrow i} \sum _{T \in T_{i'}} \prod _{(j \rightarrow j') \in S} k_{j \rightarrow j'} \\&= \sum _{(i' \rightarrow i) \in G} k_{i' \rightarrow i} \, (K_k)_{i'} . \end{aligned}$$

Hence,

$$\begin{aligned} \sum _{(i' \rightarrow i) \in G} k_{i' \rightarrow i} \, (K_k)_{i'} - \sum _{(i \rightarrow i') \in G} k_{i \rightarrow i'} \, (K_k)_i = 0 \quad \text {for } i \in V, \end{aligned}$$

that is, $\psi = K_k$ solves $A_k \, \psi = 0$. $\square$

### Fact 11

$$\begin{aligned} A_k {{\,\textrm{diag}\,}}(K_k) = \sum _{C} \lambda _{k,C} \, A_C, \end{aligned}$$

where the sum is over all cycles *C* contained in *G*,

$$\begin{aligned} \lambda _{k,C} = \sum _{S \in G_C} \; \prod _{(j \rightarrow j') \in S} k_{j \rightarrow j'}, \end{aligned}$$

and $A_C$ is the Laplacian matrix of the cycle *C* with $k=\bar{1} \in {\mathbb R}^E_>$ (all edge labels set to 1).

### Proof

Both matrices, $A_k {{\,\textrm{diag}\,}}(K_k)$ and $\sum _{C} \lambda _{k,C} \, A_C$, have zero row and column sums. Hence, it is sufficient to compare the off-diagonal entries.

On the one hand, every spanning tree in $T_i$ gives rise to a subgraph in $G_C$ that contains the edge $i \rightarrow i'$ in the cycle and vice versa (by adding/removing the edge $i \rightarrow i'$). For $i \ne i'$,

$$\begin{aligned} (A_k {{\,\textrm{diag}\,}}(K_k))_{i',i}&= k_{i \rightarrow i'} (K_k)_i \\&= \sum _{T \in T_i} \; \prod _{(j \rightarrow j') \in T} k_{j \rightarrow j'} \cdot k_{i \rightarrow i'} \\&= \sum _{C :(i \rightarrow i') \in C} \sum _{S \in G_C} \prod _{(j \rightarrow j') \in S} k_{j \rightarrow j'} \\&= \sum _{C :(i \rightarrow i') \in C} \lambda _{k,C} . \end{aligned}$$

On the other hand,

$$\begin{aligned} (A_C)_{i',i} = {\left\{ \begin{array}{ll} 1, &{}\quad \text {if } (i \rightarrow i') \in C, \\ -1, &{}\quad \text {if } i = i', \\ 0, &{}\quad \text {otherwise.} \end{array}\right. } \end{aligned}$$

Hence,

$$\begin{aligned} \left( \sum _{C} \lambda _{k,C} \, A_C \right) _{i',i}&= \sum _{C :(i \rightarrow i') \in C} \lambda _{k,C} , \end{aligned}$$

and the two matrices, $A_k {{\,\textrm{diag}\,}}(K_k)$ and $\sum _{C} \lambda _{k,C} \, A_C$, agree. $\square$

*Remark* In a time-discrete, linear process $\psi ' = B_k \, \psi$ with

$$\begin{aligned} (B_k)_{i,j} = {\left\{ \begin{array}{ll} k_{j \rightarrow i}, &{}\quad \text {if } (j \rightarrow i) \in E, \\ 1-\sum _{(i \rightarrow i') \in E} k_{i \rightarrow i'}, &{}\quad \text {if } i = j, \\ 0, &{}\quad \text {otherwise,} \end{array}\right. } \end{aligned}$$

the edge labels $k \in {\mathbb R}^n_>$ do not represent transition rates, but transition probabilities. Then, $\sum _{(i \rightarrow i') \in E} k_{i \rightarrow i'} \le 1$, and $B_k$ is simply the matrix of transition probabilities with “$k_{i \rightarrow i}$”$=1 - \sum _{(i \rightarrow i') \in E} k_{i \rightarrow i'}$ and column sums equal to one. That is, $B_k = A_k + \textrm{I}$, the identity matrix. Obviously, $\psi = B_k \, \psi$ if and only if $A_k \, \psi = 0$. Whereas $A_k {{\,\textrm{diag}\,}}(K_k)$ always has zero row and column sums, $B_k$ may (or may not) be doubly stochastic (have column *and* row sums equal to one).

The Birkhoff/von Neumann Theorem (Birkhoff 1946; von Neumann 1953) states that every doubly stochastic (d.s.) matrix $B \in {\mathbb R}^{n \times n}_\ge$ is the convex sum of permutation matrices; however, this decomposition is not unique. In fact, there are *n*! permutation matrices. Still, the polytope of d.s. matrices lies in an $(n-1)^2$-dimensional affine subspace of ${\mathbb R}^{n \times n}_\ge$, and hence, every d.s. matrix can be written as the sum of at most $(n-1)^2+1$ permutation matrices.

On the contrary, the matrix $A_k {{\,\textrm{diag}\,}}(K_k)$ is the *unique* sum of *all* Laplacian matrices of cycles. However, there are more than $(n-1)^2+1$ cycles, in general.

## B Auxiliary Graph-Theoretic Results

### Lemma 12

(cf. Feinberg and Horn 1977, Lemma 2) Let $G_k=(V,E,k)$ be a connected, labeled, simple digraph with one absorbing strong component, and $A_k$ and $I_E$ be the corresponding Laplacian and incidence matrices. Then,

$$\begin{aligned} {{\,\textrm{im}\,}}(A_k) = {{\,\textrm{im}\,}}(I_E). \end{aligned}$$

### Proof

From graph theory, we know that $\dim {{\,\textrm{im}\,}}(I_E) = |V|-1$ and $\ker (A_k) = {{\,\textrm{im}\,}}\xi$, where $\xi \in {\mathbb R}^V_\ge$ has support on the absorbing strong component of *G*. Hence, also $\dim {{\,\textrm{im}\,}}(A_k) = |V|-1$. By definition, ${{\,\textrm{im}\,}}(A_k) \subseteq {{\,\textrm{im}\,}}(I_E)$ and hence, ${{\,\textrm{im}\,}}(A_k) = {{\,\textrm{im}\,}}(I_E)$. $\square$

### Lemma 13

(cf. Müller and Regensburger 2014, Proposition 5) Let $G=(V,E)$ be a connected, simple digraph, $G_\mathcal {E}= (V,\mathcal {E})$ be an auxiliary digraph, and $I_E$ and $I_\mathcal {E}$ be the corresponding incidence matrices. Then,

$$\begin{aligned} {{\,\textrm{im}\,}}(I_\mathcal {E}) = {{\,\textrm{im}\,}}(I_E). \end{aligned}$$

### Proof

From graph theory and the definition of an auxiliary graph, we know that $\dim {{\,\textrm{im}\,}}(I_E) = \dim {{\,\textrm{im}\,}}(I_\mathcal {E}) = |V|-1$. In the rest of the proof, we show that ${{\,\textrm{im}\,}}(I_E) \subseteq {{\,\textrm{im}\,}}(I_\mathcal {E})$. We consider the edge $(i \rightarrow j) \in E$ and the corresponding column $e^j-e^i$ of $I_E$, where $e^i$ denotes the *i*th standard basis vector in ${\mathbb R}^V$. Since $G_\mathcal {E}$ is a directed tree, there is a path from *i* to *j* in the undirected version of $G_\mathcal {E}$, that is, $i = i_1 -\hspace{-2ex}-\;i_2 -\hspace{-2ex}-\;\ldots -\hspace{-2ex}-\;i_l = j$ with either $(i_k \rightarrow i_{k+1}) \in \mathcal {E}$ or $(i_k \leftarrow i_{k+1}) \in \mathcal {E}$ for $k=1,\ldots ,l-1$. Hence,

$$\begin{aligned} e^j-e^i = \sum _{k=1}^{l-1} \alpha _k \left( e^{i_{k+1}} - e^{i_k} \right) , \end{aligned}$$

where $\alpha _k \in \{-1, 1\}$ and $e^{i_{k+1}} - e^{i_k}$ is the column of $I_\mathcal {E}$ corresponding to either the edge $(i_k \rightarrow i_{k+1}) \in \mathcal {E}$ or $(i_k \leftarrow i_{k+1}) \in \mathcal {E}$. $\square$

## C A Proof of Theorem 5

We provide a proof of Theorem 5 in the main text, based on the entropy-like Lyapunov function. Previous proofs further use inequalities for the exponential function or the logarithm and cycle decomposition of the graph, cf. Horn and Jackson (1972), Sontag (2001), Anderson (2014) and Gopalkrishnan (2014). We use monomial evaluation orders and corresponding geometric objects (strata and polyhedral cones).

### Theorem

Let $(G_k,y)$ be a mass-action system and $x^* \in {\mathbb R}^n_>$ be a positive CBE of the dynamical system (4). Then,

$$\begin{aligned} \left( \ln \frac{x}{x^*} \right) ^\textsf{T}\! f_k(x) < 0 \end{aligned}$$

for all $x \in {\mathbb R}^n_>$ that are not complex-balanced equilibria. Hence, (i) all positive equilibria are complex-balanced, and (ii) $x^*$ is asymptotically stable.

### Proof

Let $x \in {\mathbb R}^n_>$ not be a CBE. Then, there is a full-dimensional subset (a stratum) $\mathcal {S}_{k,\mathcal {E}}\subset {\mathbb R}^n_>$ for some chain graph $G_\mathcal {E}=(V,\mathcal {E})$ such that $x \in \mathcal {S}_{k,\mathcal {E}}$, that is, $\ln \frac{x}{x^*} \in \mathcal {C}_\mathcal {E}$.

Using the dynamical system (4) and Theorem 4, we have

$$\begin{aligned} \left( \ln \frac{x}{x^*} \right) ^\textsf{T}\! f_k(x)&= \left( \ln \frac{x}{x^*} \right) ^\textsf{T}Y A_k \, x^Y \\&= - \left( \ln \frac{x}{x^*} \right) ^\textsf{T}Y I_\mathcal {E}\mathcal {A}_{k,\mathcal {E}}I_\mathcal {E}^\textsf{T}{{\,\textrm{diag}\,}}(K_k^{-1}) \, x^Y \\&= - \, a^\textsf{T}\mathcal {A}_{k,\mathcal {E}}\, b \end{aligned}$$

with

$$\begin{aligned} a(x)&= (Y I_\mathcal {E})^\textsf{T}\ln \frac{x}{x^*} , \\ b(x)&= I_\mathcal {E}^\textsf{T}{{\,\textrm{diag}\,}}(K_k^{-1}) \, x^Y . \end{aligned}$$

Using $\mathcal {S}_{k,\mathcal {E}}$ and $\mathcal {C}_\mathcal {E}$ as in Eqs. (8) and (10), we have $b \ge 0$ and $a \ge 0$.

Since *x* is not be a CBE, $b \ne 0$, that is, there is $i \rightarrow i' \in \mathcal {E}$ such that

$$\begin{aligned} b_{i \rightarrow i'} = \frac{x^{y(i')}}{(K_k)_{i'}} - \frac{x^{y(i)}}{(K_k)_i} > 0. \end{aligned}$$

By complex balancing (6),

$$\begin{aligned} \left( \frac{x}{x^*}\right) ^{y(i')} - \left( \frac{x}{x^*}\right) ^{y(i)} > 0 \end{aligned}$$

and hence also

$$\begin{aligned} a_{i \rightarrow i'} = (y(i')-y(i))^\textsf{T}\ln \frac{x}{x^*} > 0. \end{aligned}$$

By Theorem 4, the core matrix of the graph Laplacian, $\mathcal {A}_{k,\mathcal {E}}\in {\mathbb R}^{\mathcal {E}\times \mathcal {E}}$ is non-negative with positive diagonal. Hence,

$$\begin{aligned} \left( \ln \frac{x}{x^*} \right) ^\textsf{T}\! f_k(x) = - a^\textsf{T}\mathcal {A}_{k,\mathcal {E}}\, b < 0. \end{aligned}$$

(i) If there is a positive equilibrium $x \in {\mathbb R}^n_>$ that is not complex-balanced, then $f_k(x)=0$, contradicting $\left( \ln \frac{x}{x^*} \right) ^\textsf{T}\! f_k(x) < 0$.
(ii) Recall that a positive CBE $x^*$ is the unique steady state in its stoichiometric compatibility class (forward invariant set). Hence,
  $$\begin{aligned} \frac{\textrm{d} }{\textrm{d} t} \, L(x(t)) = \left( \ln \frac{x}{x^*} \right) ^\textsf{T}\! f_k(x) \le 0 \quad \text { with ``='' if and only if } x=x^*, \end{aligned}$$
  and *L*(*x*) is a strict Lyapunov function. $\square$
